# Cluster analysis of insomnia symptoms during COVID-19 pandemic: a general population web-based survey in Iran

**DOI:** 10.5935/1984-0063.20200087

**Published:** 2021

**Authors:** Khosro Sadeghniiat-Haghighi, Mohammad-Mehdi Mehrabinejad, Arezu Najafi, Mahya Shabani, Samaneh Akbarpour

**Affiliations:** 1 Tehran University of Medical Sciences, Occupational Sleep Research Center, Baharloo Hospital, Tehran University of Medical Sciences - Tehran - Iran.; 2 Tehran University of Medical Sciences, Students’ Research Committee - Tehran - Iran.

**Keywords:** Fear, Sleep Initiation and Maintenance Disorders, Coronavirus Infections, Sleep, SARS-CoV-2

## Abstract

**Objective:**

To investigate the prevalence of insomnia and its different phenotypes as well as their association with fear of COVID-19 in the general population.

**Material and Methods:**

This was a cross-sectional study conducted using an online survey (e-poll). All available participants who completed the online survey form were included in the current study. All individuals with a history of sleep problems were excluded. A questionnaire package consisted of insomnia severity index (ISI), and FCV-19 for corona fear was administered for all participants. Insomnia was defined as ISI≥8. Insomnia phenotypes were considered as: (a) DIS: difficulty initiating sleep; (b) DMS: difficulty maintaining sleep; (c) EMA: early morning awakening; and (d) combined insomnia.

**Results:**

A total of 1,223 participants [827 (67.6%) female, mean age=39.82±10.75 years old], enrolled in the current survey. Based on ISI, 675 (55.2% [95%CI=52.40-57.98]) were categorized into the insomnia group. Insomnia was more prevalent in females (p=0.006), participants with 50 years old or higher (p=0.04), or high fear of COVID-19 (p<0.0001). Totally, 67.4%, 66.4%, and 55% of all participants had DIS, DMS, and EMA, respectively, in the current outbreak. Besides, 79% had impaired daily functioning, 51.6% had impaired quality of life, and 62% were worried about their sleep problem. Notably that a considerable percentage of individuals with normal ISI scores had at least one insomnia phenotype or impaired daily functioning and quality of life. Further analyses revealed a significant increasing trend in all four insomnia phenotypes prevalence with an increase in fear of COVID-19 (all p-values<0.0001).

**Conclusion:**

Individuals with higher age, female gender, or higher fear of COVID-19 are at higher risk of all types of insomnia as well as impaired daytime performance or quality of life.

## INTRODUCTION

Coronavirus infectious disease 2019 (COVID-19) caused by severe acute respiratory syndrome coronavirus-2 (SARS-CoV-2) was first reported in late December 2019, in Wuhan, China. This new infectious disease, with the etiology of a single-strand ribonucleic acid virus (a member of the *Coronaviridae* family)^1^, has an unignorably debilitating impact on global health. Given its extremely contagious nature, the World Health Organization (WHO) announced the outbreak of a public health emergency of international concern on January 30^th^, 2020^1^.

Although COVID-19 with somatic complications could eventuate in high morbidity and mortality worldwide, the neuro-psychiatric consequences could affect more individuals in the general population even with no COVID-19 symptoms and might remain in the long-term if no measure is taken^2^. With the extremely high contagious nature and relatively high morbidity and mortality, fear and worrying in general population could be increased resulting in psychosocial problems^3^. Fear of COVID-19, economic problems, large-scale quarantine, and excessive use of social media applications and electronic devices might result in the development or exacerbation of various psychological disorders, including sleep problems^4^. Beside the adverse effects of sleep problems on individuals’ daily performance and quality of life, some critical psychological issues, including interpersonal and intrapersonal challenges and accordingly homicidal or suicidal ideas, are significantly correlated with insomnia^5,6^.

Not only the adverse effects of the COVID-19 pandemic itself but also national and regional policies in controlling this outbreak could impact the mental health among the general population^7^. Those psychological disorders, including sleep problems, could interfere with their daily functioning and impair their quality of life and even reduces immune function and make them more vulnerable to COVID-19^8,9^. Although the psychological consequences of the COVID-19 outbreak, particularly in healthcare workers, were widely discussed, the four insomnia phenotypes and their association with fear of COVID-19 in the general population were less investigated^10,11^. Hence, in the present study, we investigated the prevalence of insomnia and its different phenotypes as well as their association with fear of COVID-19 in the general population using a nationwide web-based survey.

## MATERIAL AND METHODS

### Study design and participants

This cross-sectional study was conducted using an online survey (e-poll) to avoid direct contact with participants during the ongoing crisis of COVID-19. The study was approved in the Research Deputy of Tehran University of Medical Sciences (IR.TUMS.VCR.REC.1399.233). This study conducted in concordance with the World Medical Association Declaration of Helsinki and approved by the ethics committee of the Tehran University of Medical Sciences. Prior to the administration of questionnaires, the nature and goals of the study informed to all the participants and they were assured that their responses would be confidential to the research team.

The sampling method was available participants who completed the online survey form. The all available social media was used for distributing the questionnaires. The questionnaire accompanied a short description of the project’s objectives, and the participants were asked to complete the online questionnaire. All individuals with a history of sleep problems were excluded. Totally, 1,223 participants (827 (67.6%) females) participated in the current survey.

### Instruments: Insomnia severity index (ISI)

ISI comprises seven questions: difficulty falling asleep, difficulty maintaining sleep, early morning awakening, subject’s satisfaction from own sleep, the influence on subject’s life, and concern and amount of influence on subject’s daily life. Each question has scores of 0-4. Total ISI score is categorized as follows: 0-7: normal, 8-14 mild insomnia, 15-21: moderate clinical insomnia, 22-28: severe clinical insomnia^12^. Insomnia was defined as ISI≥8.

Insomnia phenotypes were considered as: (a) DIS: difficulty initiating sleep; (b) DMS: difficulty maintaining sleep; (c) EMA: early morning awakening; and (d) combined insomnia.

### FCV-19 for corona fear

FCV-19 has 7-Likert scale items with scores ranging from ‘1: strongly disagree’ to ‘5: strongly agree’. FCV-19 questions include: afraid of COVID-19 severity, being uncomfortable and hands become clammy when thinking about COVID-19, being afraid of losing life, being anxious when reading about COVID-19 news, and sleep problems and palpitation due to COVID-19.

The total score is the sum of all items’ scores (7-35) and higher scores indicate higher fear of COVID-19. Validation of this questionnaire is done by Ahorsu et al. (2020)^13^ in Iran.

### Statistical analysis

The completed data was exported by the excel software Microsoft Office Version. Stata (StataCorp. 14 SE) was used to analyze data, and *p*-value<0.05 was considered statistically significant. The study population characteristics are presented as mean±SD or frequency and percent for continuous and qualitative variables, respectively. The differences in characteristics were examined using the chi-square test for categorical variables and student’s independent t-test for continuous variables. Nearly 1.74% of data cells had missing values. Therefore, the unknown (missing) values were imputed by the regression model and single imputation method in mice package using R software^14,15^.

Crude and multiple logistic regression models were used to estimate the odds ratio (OR) with 95% confidence intervals (95%CI) of different risk factors for the development of interested outcomes. To assess the trends of insomnia prevalence and each subscale, we generated quantile boundaries for the distribution of fear of the COVID-19 scale and determined the insomnia prevalence and all seven subscales in each quantile.

## RESULTS

A total of 1,223 participants, consisted of 827 (67.6%) females with the mean age of all participant=39.82±10.75 years old (Table 1), enrolled in the current survey. Of those, 844 (69%) were married, 505 (41.3%) were self-employment, and 764 (62.5%) were upper diploma.

**Table 1:** Socio-demographic characteristics of Iranian online survey-2020 based on insomnia.

	Total (n=1223)	Non-insomnia (n=548)	Insomnia (n=675)	p-value
Age, year (Mean (SD))	39.82 (10.75)	39.24±10.21	40.28±11.15	0.093
Fear of COVID-19 score (Mean (SD))	19.70 (5.08)	17.69±4.35	21.33±5.04	<0.0001
	**Percent (95%CI)**	**Percent (95%CI)**	**Percent (95%CI)**	
Gender				
Male	32.37 (29.76-35.00)	36.49 (32.45-40.53)	29.03 (25.60-32.46)	0.006
Female	67.62 (64.99-70.24)	63.59 ( 59.46-67.54)	71.96 (67.53-74.39)	
**Age**				
<50 years	80.45 (78.23-82.68)	83.02 (79.88-86.17)	78.37 (75.25-81.48)	0.041
50<=	19.54 (17.31-21.76)	16.97 (13.82-20.11)	21.62 (18.51-24.74)	
**Marital status**				
Married	69.01 (66.41-71.61)	70.25 (66.42-74.09)	68.00 (64.47-71.52)	0.396
Single	30.98 (28.39-33.58)	29.74 (25.90-33.57)	32.00 (28.47-35.52)	
**Occupation**				
Self-employment	41.29 (38.52-44.05)	40.69 (36.57-44.81)	41.77 (38.05-45.50)	0.903
Government employee	35.48 (32.80-38.17)	36.13 (32.10-40.16)	34.96 (31.35-38.56)	
Unemployment or student	23.22 (20.85-25.59)	23.17 (19.63-26.71)	23.25 (20.06-26.45)	
**Education**				
Under diploma	14.63 (12.65-16.61)	16.97 (13.82-20.11)	12.74 (10.22-15.26)	0.080
Diploma	22.89 (20.53-25.52)	21.16 (17.74-24.59)	24.29 (21.05-27.53)	
Upper diploma	62.46 (59.75-65.18)	61.86 (57.78-65.93)	62.96 (59.31-66.61)	

Chi-square test was uses for all categorical variables; t-student test was used for age and fear of COVID-19 as continuous variables.

Totally, the mean±SD of ISI score was 9.67±5.8 (range=0-27). Based on ISI, 675 (55.2% [95%CI=52.40-57.98]) were categorized into the insomnia group. Insomnia was more prevalent in females (*p*=0.006) and participants with 50 years old or higher (*p*=0.04) or high fear of COVID-19 (*p*<0.0001) (Table 1). There was no significant association between insomnia and marital status, employment, or education level. The details of the socio-demographic characteristics of all participant and their differences between insomniac and non-insomniac groups are presented in Table 1.

Additionally, all four insomnia phenotypes, as well as impaired daily functioning and quality of life, were significantly more common in the insomnia group (all *p*-values<0.0001) (Table 2). Notably that a considerable percentage of individuals with normal ISI scores had at least one insomnia phenotype or impaired daily functioning and quality of life (Table 2). Considering all enrolled participants as a random sample of the general population, a relatively high percentage of individuals with sleep problems that have impaired their daytime performance and quality of life is evident. Totally, 67.4%, 66.4%, and 55% of all participants had DIS, DMS, and EMA, respectively, in the current outbreak. Besides, 79% had impaired daily functioning, 51.6% had impaired quality of life, and 62% were worried about their sleep problem (Table 2). On note that combined types (DIS+DMS+EMA, DIS+DMS, DIS+EMA, and EMA+DMS) of insomnia showed the prevalence of 26.6%, 13.4%, 3.8%, and 5.0%, respectively.

**Table 2:** Details of ISI subscales in all participants and insomnia groups.

Insomnia patterns	Total (n=1223) N (percent)	Non-insomnia (n=548) N (percent)	Insomnia (n=675) N (percent)	p-value
Difficulty falling asleep	824 (67.4)	207 (37.8)	617 (91.4)	<0.0001
Difficulty staying asleep	812 (66.4)	227 (41.4)	585 (86.7)	<0.0001
Problems waking up too early	673 (55)	153 (27.9)	520 (77)	<0.0001
To what extent do you consider your sleep problem to interfere with your daily functioning (e.g., daytime fatigue, mood, ability to function at work/daily chores, concentration, memory, mood, etc.) currently?	966 (79)	313 (57.1)	653 (96.7)	<0.0001
How noticeable to others do you think your sleep problem is in terms of impairing the quality of your life?	631 (51.6)	98 (17.9)	533 (79)	<0.0001
How worried are you about your current sleep problem?	758 (62)	159 (29)	599 (88.7)	<0.0001
How satisfied are you with your current sleep pattern?	942 (77)	472 (86.1)	470 (69.6)	<0.0001

**Figure 1:**
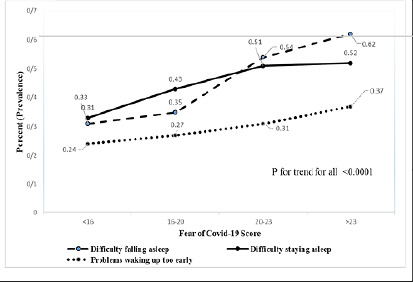
Trend of insomnia phenotype in participants without insomnia.

Further analyses to assess the correlation between fear of COVID-19 and insomnia phenotypes revealed a significant increasing trend in all four insomnia phenotypes’ prevalence with an increase in fear of COVID-19 (all *p*-values<0.0001) (Table 3 and Figure 1).

**Table 3:** Association between type of sleep disruption scores and severity of fear of COVID-19 in study individuals.

	Fear of COVID-19				
	First quartile	Second quartile	Third quartile	Fourth quartile	p-for trend
Range of score	<16	16-20	20-23	>23	
Difficulty falling asleep	0.47±0.42	0.63±0.48	0.77±0.41	0.86±0.34	<0.0001
Difficulty staying asleep	0.48±0.50	0.63±0.48	0.73±0.44	0.85±0.35	<0.0001
Problems waking up too early	0.39±0.32	0.50±0.24	0.60±0.49	0.74±0.43	<0.0001
To what extent do you consider your sleep problem to interfere with your daily functioning (e.g., daytime fatigue, mood, ability to function at work/daily chores, concentration, memory, mood, etc.) currently?	0.67±0.46	0.76±0.42	0.76±0.42	0.85±0.34	<0.0001
How noticeable to others do you think your sleep problem is in terms of impairing the quality of your life?	0.34±0.27	0.47±0.49	0.56±0.49	0.73±0.44	<0.0001
How worried are you about your current sleep problem?	0.44±0.39	0.55±0.49	0.70±0.45	0.83±0.37	<0.0001
How satisfied are you with your current sleep pattern?	0.81±0.38	0.82±0.37	0.74±0.43	0.67±0.47	<0.0001

Data are reported as mean ± SD.

## DISCUSSION

Stress level rises during a pandemic as people are worried about their health condition and financial results that may affect their sleep adversely. Besides, behavioural and routine daily life changes due to pandemic including long-term home isolation, distance working, lower physical activities, exposure to sunlight, excessive use of multimedia devices, and changes in social life could impact sleep homeostasis^11^. These two factors – increase of stress level and behavioural and routine daily life changes – can significantly infuence each three sleep regulatory processes: the circadian rhythm, the homeostatic sleep drive, and the arousal system^16^. Our findings revealed the prevalence of clinical insomnia (ISI≥8) as 55.2%. Insomnia prevalence was higher than reported values in previous surveys, which was probably due to differences in cut-off values and utilized questionnaire. For instance, Voitsidis et al. (2020)^11^ used Athens insomnia scale (AIS) and reported about 37% insomnia in Greek population^11^. In another study on 556 adult participants, almost 19% were categorized in clinical insomnia (ISI≥15)^10^. Finally, another national survey in Italy considered ISI≥22 as severe insomnia and 7.3% of participants met the criteria^17^.

We observed that insomnia was more prevalent among participants with female gender, age≥50, or higher fear of COVID-19. Besides, all four insomnia phenotypes (DIS, DMS, and EMA), as well as impaired daily functioning and quality of life, had a significantly positive association with fear of COVID-19. Our findings are compatible with ample evidence suggesting that females are more vulnerable to stress-related disorders like anxiety disorders or post-traumatic stress disorder^18^. In line with our findings, previous studies on the general population reported the insomnia prevalence as 29.3% and 37.6% with the dominancy of females^11,19^.

Another national web-based sur vey on 18147 participants reported higher severe insomnia prevalence among females^17^.

Furthermore, higher-aged individuals, however, have more underlying diseases, making them more prone to COVID-19, consequently more stressed about being infected.

Additionally and of note, non-insomniac individuals were not immune from sleep problems in the current outbreak and accordingly impaired daily functioning and quality of life, highlighting the role of general education and large-scale measures to minimize the possible long-term sequels. During each outbreak, people usually seek-out the related information to protect themselves, and when regular information from official sources is lacking, they may rely on conficting information disseminated from social media leading to higher stress^20^. Hence, global health measures should be taken to reduce the psychological stressors and false information by strictly controlling social media outputs. Besides, previous studies have posed recommendations for regulating sleep during this outbreak. Recommendations mainly include adopting environmental and behavioural controls, reducing maladaptive coping approaches (e.g., alcohol), regular bed/wake time schedule, being physically active and shortly exposed to sunlight, and limit exposure to conficting and false news^21^.

Last but not least, our findings should be interpreted in light of some limitations. Respondents’ unavailability, lack of interviewer, possible cooperation problems, respondents’ lack of online experience, and using smartphones by higher educated individuals are several limitations of online surveys. By using a standard sleep questionnaire in the local language, we tried to reduce the limitations of the study. Further investigations using in-person interviews are recommended to address this critical issue.

In conclusion, in the current pandemic, individuals with higher age, female gender, or higher fear of being infected were at higher risk of all types of insomnia (DIS, DMS, or EMA) as well as impaired daytime performance or quality of life. Seemingly, healthy individuals also showed relatively high insomnia rates, which had interfered with their daily functioning. To avoid the psychosocial consequences of impaired sleep health, large-scale measures should be employed to mitigate or prevent the short-term or long-term consequences of the pandemic on sleep health.

## Appendix

**Supplementary Table:** Insomnia among Iranian population at the item level (n=1223) ISI>8.

Insomnia items in population		None	Mild	Moderate	Sever	Very severe
Difficulty falling asleep	Total population (n=1223)	93 (7.6)	204 (16.7)	395 (32.3)	441 (36.1)	90 (7.4)
	Insomnia (n=675)	40 (5.9)	80 (11.9)	205 (30.4)	287 (42.5)	63 (9.3)
	Non-insomnia(n=548)	53 (9.7)	124 (22.6)	190 (34.7)	154 (28.1)	27 (4.9)
Difficulty staying asleep	Total population		284 (23.2)	273 (22.3)	576 (47.1)	90 (7.4)
	Insomnia		108 (16)	151 (22.4)	349 (51.7)	67 (9.9)
	Non-insomnia		176 (32.1)	122 (22.3)	227 (41.4)	23 (4.2)
Problems waking up too early	Total population	0	915 (74.8)	146 (11.9)	150 (12.3)	12 (1)
	Insomnia	0	433 (64.1)	106 (15.7)	127 (18.8)	9 (1.3)
	Non-insomnia	0	282 (88)	40 (7.3)	23 (4.2)	3 (0.5)
	**Not at all**	**A little**	**Somewhat**	**Much**	**Very much**
To what extent do you consider your sleep problem to interfere with your daily functioning (e.g. daytime fatigue, mood, ability to function at work/daily chores, concentration, memory, mood, etc.) currently?	Total population	145 (11.9)	241 (19.7)	251 (20.5)	437 (35.7)	149 (12.2)
	Insomnia	64 (9.4)	106 (15.7)	144 (21.3)	263 (39)	98 (14.5)
	Non-insomnia	81 (14.8)	135 (24.6)	107 (19.5)	174 (31.8)	51 (9.3)
How noticeable to others do you think your sleep problem is in terms of impairing the quality of your life?	Total population	102 (8.3)	235 (19.2)	328 (26.8)	468 (38.3)	90 (7.4)
	Insomnia	38 (5.6)	88 (13)	172 (25.5)	306 (45.3)	71 (10.5)
	Non-insomnia	64 (11.7)	147 (26.8)	156 (28.5)	162 (29.6)	19 (3.5)
How worried are you about your current sleep problem?	Total population	424 (34.7)	425 (34.8)	181 (14.8)	174 (14.2)	19 (1.6)
	Insomnia	146 (21.6)	228 (33.8)	139(20.6)	145 (21.5)	17 (2.5)
	Non-insomnia	278 (50.7)	197 (35.9)	42 (7.7)	29 (5.3)	2 (0.4)
How satisfied are you with your current sleep pattern?	Total population	356 (29.1)	440 (36)	200 (16.4)	200 (16.4)	27 (2.2)
	Insomnia	132 (19.6)	219 (32.4)	144 (21.3)	158 (23.4)	22 (3.3)
	Non-insomnia	224 (40.9)	221 (40.3)	56 (10.2)	42 (7.7)	5 (0.9)
